# A validated model for individualized prediction of pregnancy outcome in woman after fresh cycle of Day 5 single blastocyst transfer

**DOI:** 10.1038/s41598-023-36824-8

**Published:** 2023-06-20

**Authors:** Lei Chen, Ruyu Jiang, Yiqun Jiang, Yuting Su, Shanshan Wang

**Affiliations:** 1 Center for Reproductive Medicine and Obstetrics and Gynecology, Nanjing Drum Tower Hospital, Nanjing University Medical School, No.321, Zhongshan Road, Gulou District, Nanjing, 210008 China; 2 Center for Molecular Reproductive Medicine, Nanjing University, Nanjing, 210008 People’s Republic of China

**Keywords:** Developmental biology, Biomarkers

## Abstract

The association between the embryo quality, clinical characteristics, miRNAs (secreted by blastocysts in the culture medium) and pregnancy outcomes has been well-established. Studies on prediction models for pregnancy outcome, using clinical characteristics and miRNA expression, are limited. We aimed to establish the prediction model for prediction of pregnancy outcome of woman after a fresh cycle of Day 5 single blastocyst transfer (Day 5 SBT) based on clinical data and miRNA expression. A total of 86 women, 50 with successful pregnancy and 36 with pregnancy failure after fresh cycle of Day 5 SBT, were enrolled in this study. All samples were divided into training set and test set (3:1). Based on clinical index statistics of enrolled population and miRNA expression, the prediction model was constructed, followed by validation of the prediction model. Four clinical indicators, female age, sperm DNA fragmentation index, anti-mullerian hormone, estradiol, can be used as independent predictors of pregnancy failure after fresh cycle of Day 5 SBT. Three miRNAs (hsa-miR-199a-3p, hsa-miR-199a-5p and hsa-miR-99a-5p) had a potential diagnostic value for pregnancy failure after Day 5 SBT. The predictive effect of model combining 4 clinical indicators and 3 miRNAs (area under the receiver operating characteristic curve, AUC = 0.853) was better than models combining single 4 clinical indicators (AUC = 0.755) or 3 miRNAs (AUC = 0.713). Based on 4 clinical indicators and 3 miRNAs, a novel model to predict pregnancy outcome in woman after fresh cycle of Day 5 SBT has been developed and validated. The predictive model may be valuable for clinicians to make the optimal clinical decision and patient selection.

## Introduction

Single blastocyst transfer (SBT) is an effective method to avoid multiple pregnancies in assisted reproductive technology (ART) cycles. The success rate of SBT depends on the efficacy of the embryo selection^1^. Therefore, in order to ensure the stability of embryo transfer rate, in those pregnancies achieved with assisted reproduction, screening of high quality embryos is of great importance for successful pregnancy outcomes.

Clinically, morphologic characteristics are widely used for screening of high quality embryos. In addition, some clinical characteristics of the patients are associated with the pregnancy outcome. La Marca et al.^2^ constructed a nomogram to predict live birth through a combination of anti-mullerian hormone (AMH) and age. The result showed that sensitivity and specificity of the model were respectively 79.2 and 44.2%. By combination of embryo quality, age and basal follicle stimulating hormone (FSH), a nomogram was constructed to predict pregnancy in 1675 double embryo transfer treatment cycles^3^. It is found that these three clinical parameters are identified as significant predictors (at 5% significance level) of pregnancy. In freeze-thawed embryo transfer cycles, a simple nomogram was developed to predict the early clinical outcomes by using multiple clinical parameters^4^. In the training cohort and validation cohort, the area under the ROC curve (AUC) is respectively 0.698 and 0.699. However, it seems that these models can only be used as a simple tool to predict pregnancy outcomes. More studies of the applicability of improving prediction models will need more comprehensive.

Recently, some researchers have focused on seeking for other methods, on account of genomics to assess the embryo quality, such as miRNA expression^5–10^. MiRNAs, can be secreted by blastocysts in the culture medium, which has significant correlation with reproductive functions in females^11^. Additionally, they can reflect the real status of fertilization potential^12^. It has been found that the abundance levels of some miRNAs can predict implantation success^13^. For example, Tan et al. reported that, compared to an in vivo-fertilized group, down-regulated miR-199a-5p in IVF blastocysts was responsible for the lower developmental potential and subsequent viability^14^. Yao et al.^15^ demonstrated that miR-99a-5p is related to implantation. Similarly, in our previous study, the expression of hsa-miR-199a-3p, hsa-miR-199a-5p and hsa-miR-99a-5p were significantly decreased in the spent culture medium of blastocyst stages on day 5 in woman with pregnancy failure after fresh cycle of Day 5 SBT^16^. Thus, we assume that the combination of clinical characteristics and miRNA expression may provide a new insight for predicting pregnancy outcomes. It should be noted that studies on prediction models for pregnancy outcome, using clinical characteristics and miRNA expression, in woman undergoing fresh cycle of Day 5 SBT are limited. Therefore, a risk prediction model for pregnancy outcome in women undergoing fresh cycle of Day 5 SBT was developed and validated in this study, which may contribute to the clinicians’ optimal clinical decision and patient selection.

## Materials and methods

### Patients

Totally, 86 women, 50 with successful pregnancy and 36 with pregnancy failure after fresh cycle of Day 5 SBT, were enrolled in this study. The inclusion criteria were: (1) females under the age of 40; (2) receive Day 5 SBT; (3) undergoing traditional IVF treatment; (4) Day 3 embryo numbers meet the timing parameters; (5) On the Day 3 of menstruation, levels of follicle-stimulating hormone (FSH) are ≤ 12 IU/mL; (6) without any specific findings in the gynecological USG. Those women who met the following criteria were excluded: (1) intracytoplasmic sperm injection (ICSI) cycles; (2) with oocyte donation; (3) recurrent implantation failure; (3) gynecological problems related to this endometrium; (4) had systemic diseases. The study was conducted in accordance with the Declaration of Helsinki and was approved by the Ethics Committee of the Drum Tower Hospital Affiliated to Nanjing University School of Medicine (2019-198-1). Informed consent was obtained from all individuals.

### Screening of variables of prediction model

In our previous study, the expression of hsa-miR-199a-3p, hsa-miR-199a-5p and hsa-miR-99a-5p were significantly decreased in the spent culture medium of blastocyst stages on day 5 in woman with pregnancy failure after fresh cycle of Day 5 SBT^16^. Therefore, hsa-miR-199a-3p, hsa-miR-199a-5p and hsa-miR-99a-5p were included as variables in the prediction model. The relative expression (2^−ΔΔCT^) of above 3 miRNAs in the blastocyst culture medium was calculated for diagnostic analysis. The pROC in R package was applied to calculate the AUC of 3 miRNAs. In addition, the compareGroups in R package was used to calculate clinical data of enrolled 86 women in T test. According to the calculation results of clinical data, 4 clinical data were selected to build the prediction model with miRNAs, including the female age, sperm DNA fragmentation index (DFI), anti-mullerian hormone (AMH), and estradiol (E_2_). In addition, the Corrplot in R package was used to calculate pearson correlation of the above 4 clinical characteristics.

### Constructing and validation of prediction model

All samples were divided into training set and test set (3:1). In the training set, logistic regression was used to build a prediction model. Logistic regression models of clinical data, miRNA and clinical data + miRNA were used for comparison. The ROC curve was drawn by epiDisplay in R package. In the test set, logistic regression models of clinical data, miRNA and clinical data + miRNA were also used for comparison. In addition, six common evaluation indicators were calculated to evaluate the performance of a prediction model, including accuracy, sensitivity, specificity, precision, recall, and F1. The bar charts were draw by the GGploT2 in R package.

### Statistical analysis

The Student’s *t* test (*t* test) in the R package (R 4.1.0) was used for the statistical analysis of clinical characteristics between pregnancy failure and successful pregnancy groups. *P* value < 0.05 is considered as statistically different.

## Results

### Baseline characteristic

Totally, 86 women, 50 with successful pregnancy and 36 with pregnancy failure after fresh cycle of Day 5 SBT, were enrolled. Clinical features of these individuals are shown in Table 1. Compared with women with successful pregnancy, the level of AMH (*P* = 0.046), the relative expression of miR-99a-5p (*P* = 0.729), miR-199a-3p (*P* < 0.001) and miR-99a-5p (*P* = 0.006) were significantly decreased in women with a pregnancy failure, while the level of bE2 was remarkably increased (*P* = 0.006). The remaining indicators were similar between the two groups (Table 1). Besides, the baseline characteristic of individuals in training set and test set were depicted in Table 2. There was no statistical difference in all indicators between the two analysis sets, except for oocytes number (*P* = 0.007), AMH level (*P* = 0.021).

**Table 1:** Baseline characteristic of enrolled 86 women.

Clinical indicators, Mean ± SD/No. (%)	Pregnancy failure (n = 36)	Successful pregnancy (n = 50)	*P* value
Maternal age (year)	30.7 ± 4.37	30.5 ± 3.58	0.838
Infertility (year)	2.85 ± 1.60	2.98 ± 2.13	0.742
Oocytes number	12.4 ± 5.44	11.5 ± 3.14	0.383
Disease type			0.827
Secondary	17 (47.2)	26 (52.0)	
Primary	19 (52.8)	24 (48.0)	
Paternal age (year)	30.8 ± 3.42	30.7 ± 3.68	0.979
Sperm volume (mL)	2.60 ± 0.80	2.43 ± 0.73	0.317
Sperm concentration (million/mL)	51.7 ± 17.7	44.7 ± 22.5	0.116
Sperm DFI	0.12 ± 0.07	0.19 ± 0.38	0.174
Blastocyst grade			0.3785
Excellent	3 (8.3)	3 (6.0)	
Good	9 (25.0)	21 (42.0)	
Average	21 (58.4)	21 (42.0)	
Poor	3 (8.3)	5 (10.0)	
NSMR	0.06 ± 0.04	0.08 ± 0.12	0.293
GnDays (day)	12.3 ± 2.46	12.1 ± 2.51	0.716
GnTotal (IU)	1997 ± 732	1949 ± 698	0.761
AMH (ng/mL)	3.37 ± 1.77	4.21 ± 2.03	0.046
BMI (kg/m^2^)	21.9 ± 2.47	23.2 ± 4.19	0.063
bFSH (mIU/mL)	7.03 ± 1.16	6.52 ± 1.62	0.096
bLH (mIU/mL)	5.52 ± 2.14	5.26 ± 2.22	0.584
bE2 (pg/mL)	46.3 ± 31.2	30.4 ± 10.5	0.006
bPRL (ng/mL)	29.7 ± 53.8	28.3 ± 66.9	0.913
bT (ng/mL)	0.28 ± 0.18	0.80 ± 4.04	0.371
EMT (mm)	11.8 ± 2.63	11.9 ± 2.27	0.930
Relative miRNA expression			
miR-199a-5p	0.20 ± 0.35	1.01 ± 2.13	0.012
miR-199a-3p	0.22 ± 0.30	0.73 ± 0.90	< 0.001
miR-99a-5p	0.43 ± 0.38	0.95 ± 1.21	0.006

**Table 2:** Baseline characteristic of test set and training set.

Clinical indicators, Mean ± SD/No. (%)	Test set	Training set	*P* value
	(n = 21)	(n = 65)	
Maternal age (year)	29.8 ± 3.68	30.9 ± 3.97	0.265
Infertility (year)	2.81 ± 1.65	2.96 ± 2.00	0.731
Oocytes number	10.0 ± 3.15	12.5 ± 4.40	0.007
Disease type			0.132
Secondary	14 (66.7)	29 (44.6)	
Primary	7 (33.3)	36 (55.4)	
Paternal age (year)	30.1 ± 3.15	30.9 ± 3.67	0.344
Sperm volume (mL)	2.40 ± 0.73	2.53 ± 0.77	0.497
Sperm concentration (million/mL)	48.2 ± 24.0	47.4 ± 19.9	0.893
Sperm DFI	0.13 ± 0.09	0.18 ± 0.35	0.316
Blastocyst grade			0.7941
Excellent	2 (9.5)	4 (6.2)	
Good	8 (38.1)	22 (33.8)	
Average	10 (47.6)	32 (49.2)	
Poor	1 (4.8)	7 (10.8)	
NSMR	0.06 ± 0.03	0.08 ± 0.11	0.270
GnDays (day)	12.0 ± 2.68	12.2 ± 2.43	0.746
GnTotal (IU)	2012 ± 818	1956 ± 676	0.776
AMH (ng/mL)	3.14 ± 1.39	4.09 ± 2.06	0.021
BMI (kg/m^2^)	23.2 ± 3.41	22.5 ± 3.69	0.446
bFSH (mIU/mL)	7.00 ± 1.13	6.65 ± 1.55	0.279
bLH (mIU/mL)	4.80 ± 2.27	5.55 ± 2.13	0.192
bE2 (pg/mL)	35.8 ± 20.4	37.6 ± 24.0	0.738
bPRL (ng/mL)	34.7 ± 70.5	27.0 ± 58.6	0.654
bT (ng/mL)	0.26 ± 0.14	0.69 ± 3.53	0.334
EMT (mm)	11.9 ± 1.66	11.9 ± 2.62	0.881
Relative miRNA expression			
miR-199a-5p	0.62 ± 1.12	0.69 ± 1.84	0.843
miR-199a-3p	0.46 ± 0.70	0.53 ± 0.78	0.707
miR-99a-5p	0.73 ± 1.14	0.73 ± 0.93	0.997

### Identification of variables of prediction model

**Figure 1:**
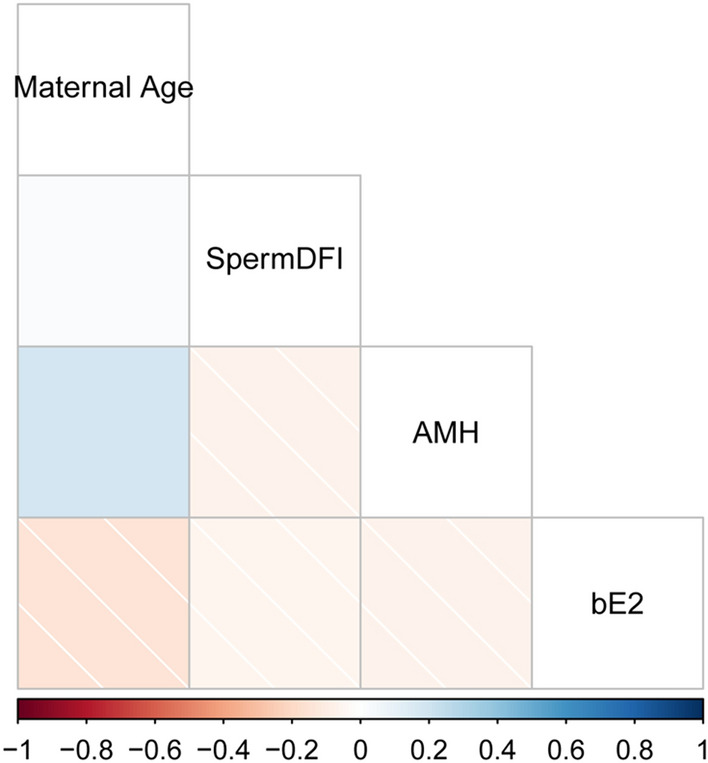
Correlation heat maps of female age, sperm DFI, AMH, and E_2_. DFI, DNA fragmentation index; AMH, anti-mullerian hormone; E_2_, estradiol.

**Figure 2:**
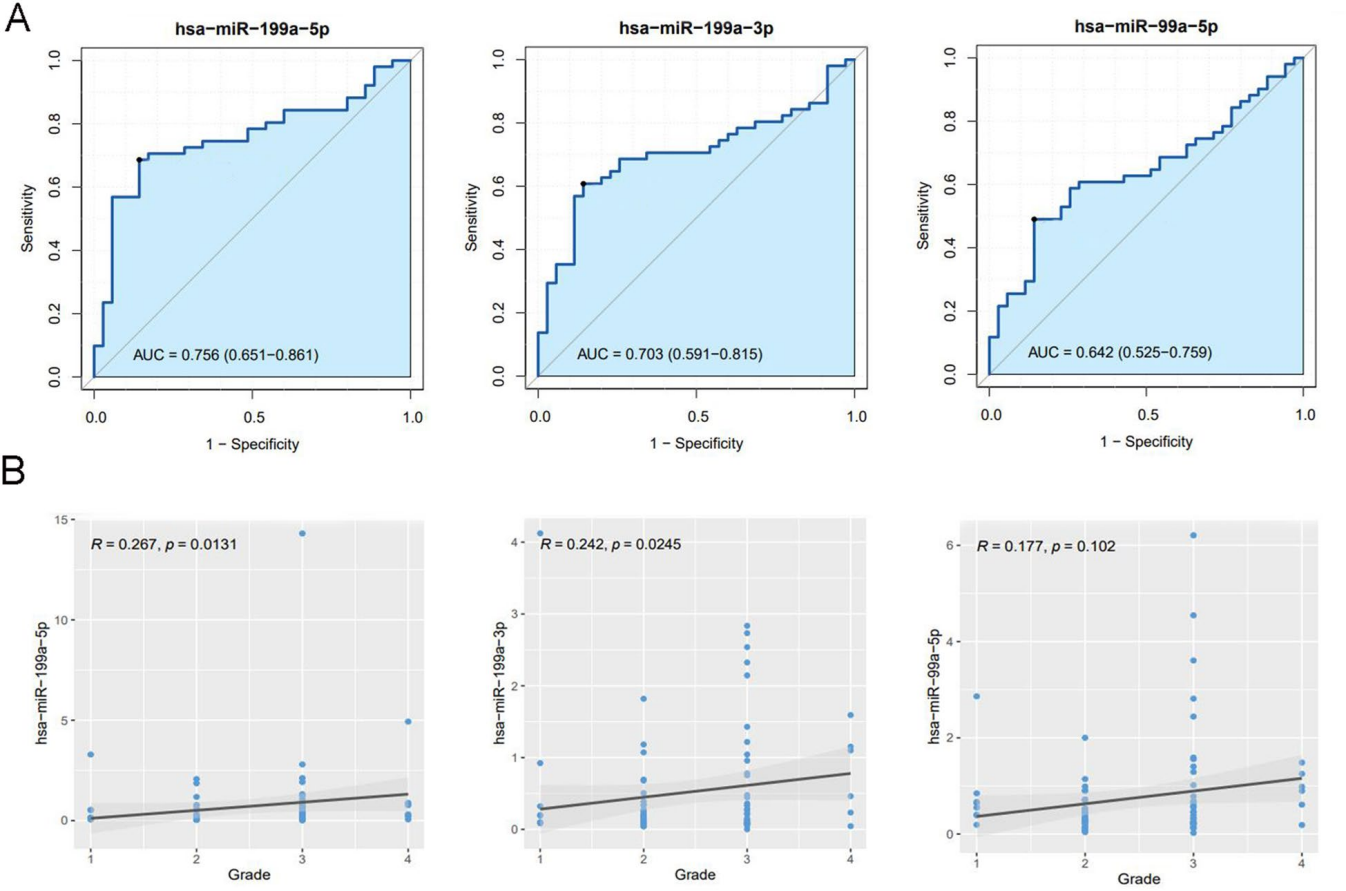
ROC analyses and correlation analysis. (**A**) ROC analyses of hsa-miR-199a-3p, hsa-miR-199a-5p and hsa-miR-99a-5p; (**B**) Correlation analysis of hsa-miR-199a-3p, hsa-miR-199a-5p and hsa-miR-99a-5p and embryo grade.

Based on clinical information, AMH and E_2_ were enrolled in the model. In addition, female age and sperm DFI were enrolled in the model according to clinical experience. There was no high correlation among the 4 clinical features (Fig. 1), indicated that these 4 clinical indicators can be used as independent predictors of pregnancy failure after fresh cycle of Day 5 SBT. In addition, hsa-miR-199a-3p, hsa-miR-199a-5p and hsa-miR-99a-5p were also enrolled in the model based on previous study^16^. Based on relative miRNA expression, the AUC values of 3 miRNAs were calculated (Fig. 2A). It is showed that the AUC values of hsa-miR-199a-3p, hsa-miR-199a-5p and hsa-miR-99a-5p were 0.703, 0.756 and 0.642, respectively. Besides, since embryonic status is important for implantation rate, we further analyzed the correlation between miRNA expression and embryo grade, and the results showed a positive correlation between the two (Fig. 2B). The above data suggested that these miRNAs had a potential diagnostic value for pregnancy failure after fresh cycle of Day 5 SBT.

### Constructing and validation of prediction model

**Figure 3:**
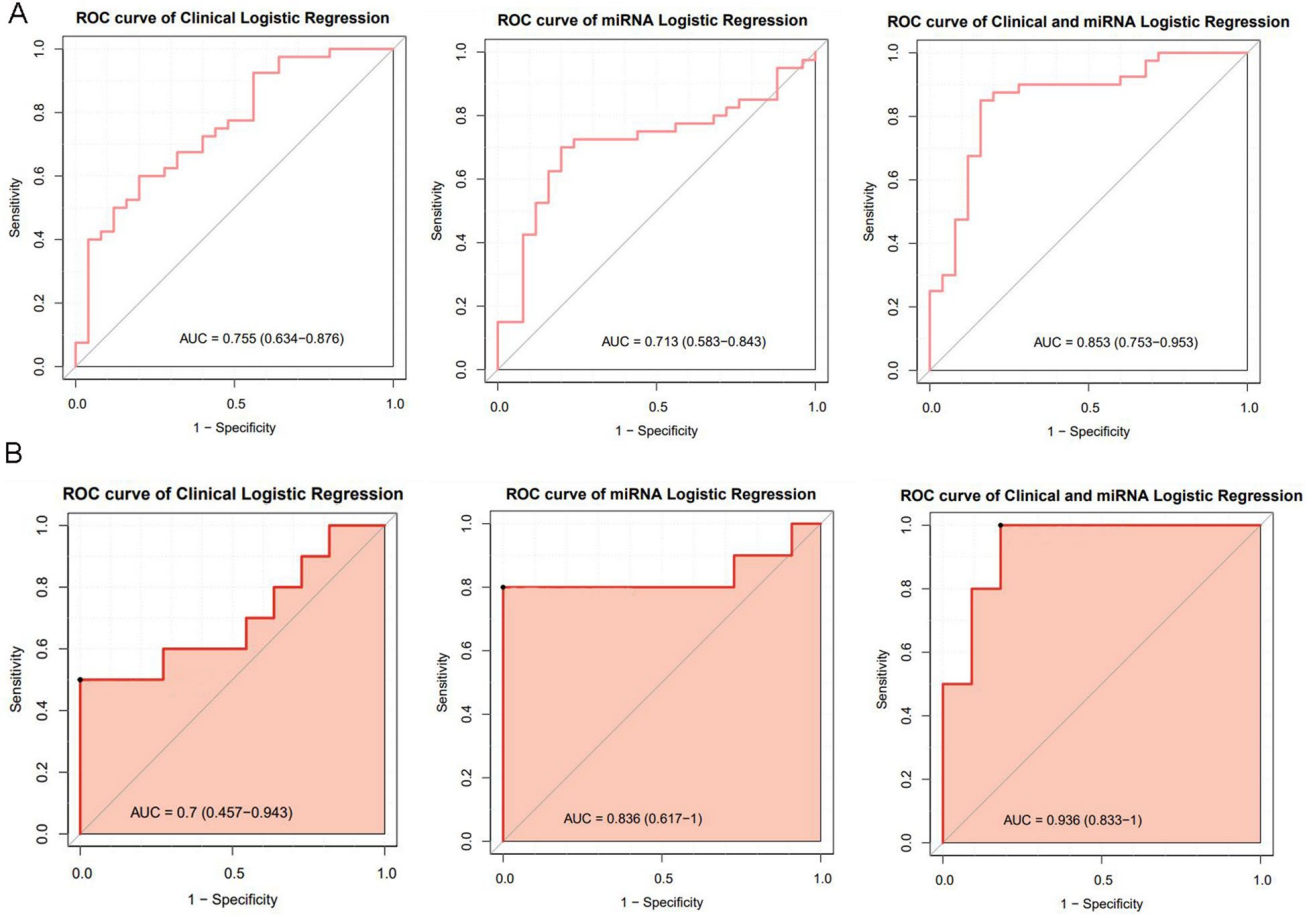
ROC analyses of 3 prediction models. (**A**) The training set; (**B**) The test set.

**Figure 4:**
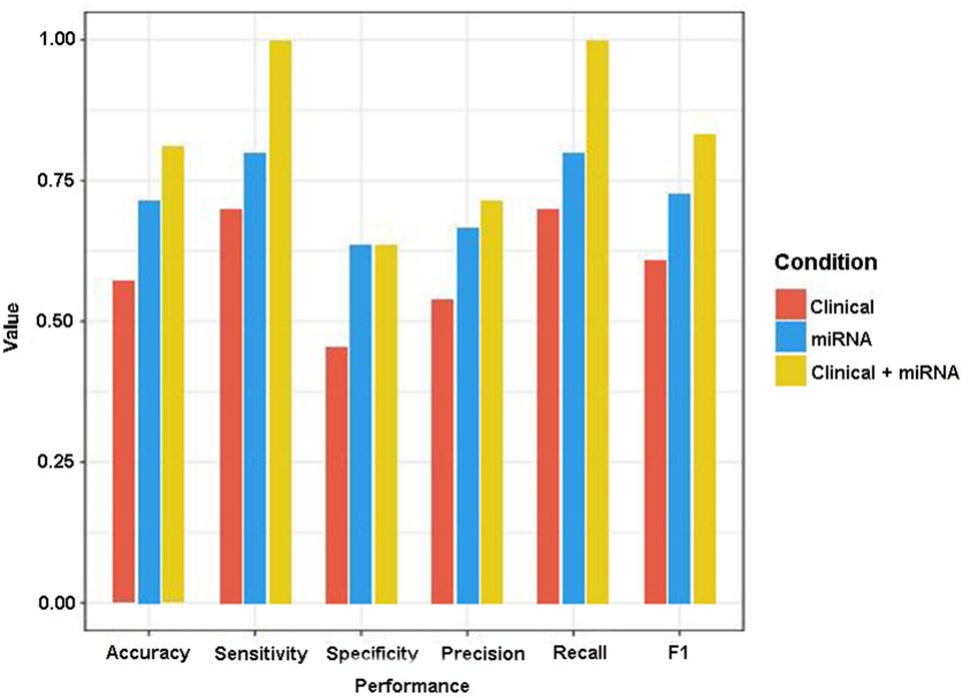
Six common evaluation indicators evaluated the performance of a prediction model.

In the training set, logistic regression models of clinical data, miRNA and clinical data + miRNA were compared. The AUC of these prediction models were 0.755, 0.713 and 0.853, respectively. In addition, the reliability of the prediction model was validated in the test set. The AUC of prediction model of clinical data, miRNA and clinical data + miRNA were 0.7, 0.836 and 0.936, respectively (Fig. 3). Statistical analysis showed that the predictive effect of model combining 4 clinical indicators and 3 miRNAs was better than models combining single 4 clinical indicators (0.853 vs. 0.755, *P* = 0.0242) or 3 miRNAs (0.853 vs. 0.713, *P* = 0.0442), but there was no statistical difference between the clinical indicators model and miRNAs model (0.713 vs. 0.755, *P* = 0.8575). Moreover, based on six common evaluation indicators (accuracy, sensitivity, specificity, precision, recall, and F1), the performance evaluation of the prediction model was validated, and the results were consistent as expected (Fig. 4).

## Discussion

Screening of high quality embryos is an important element for successful pregnancy. In addition to embryo quality, the success rate of IVF is also influenced by clinical characteristics and miRNAs expression secreted by blastocysts in the culture medium^2–4,13^. It is assumed that the combination of clinical characteristics and miRNAs expression could be used to predict pregnancy outcomes. In the present study, the prediction model was constructed for the prediction of pregnancy outcome of woman after fresh cycle of Day 5 SBT using routinely collected clinical data in hospital (female age, sperm DFI, AMH, and E_2_) and additional 3 miRNAs. The prediction model can accurately predict pregnancy outcome in women with pregnancy failure after vitro embryo transfer, with excellent diagnostic ability in internal validation.

AMH is involved in regulating early ovarian follicular growth and cyclic follicular selection^17,18^. Consistent with AMH being a strong correlate of oocyte yield, AMH has recently been proposed as a useful clinical marker for the prediction of both poor- and hyperresponses to ovarian stimulation^19^. Several authors have found a significant positive correlation between AMH concentrations and oocyte quality, fertilization rate and embryo morphology^20–22^. In addition, available data clearly indicated that AMH concentration is significantly associated with live birth and can predict the probability of live birth ^2^. For example, in Liao et al.’s study, they successfully constructed an algorithm that included AMH to predict live births^23^.The higher the AMH value, the better the ovarian reserve function. Consistently, our data showed that the clinical indicator of AMH was significantly decreased in women with pregnancy failure compared with women with successful pregnancy. Moreover, we proved that AMH can be used as an independent predictor of pregnancy failure after fresh cycle of Day 5 SBT. Collectively, the present study has substantial benefits as it demonstrates a strong predictive performance of AMH for live birth, permitting the construction of a model based this parameter.

Female age is associated with poor pregnancy outcomes^24,25^. Combing age and basal FSH, a prediction model showed a significant pregnancy rate^3^. In addition, female age combined with antral follicle count (AFC) can be helpful to estimate the pregnancy probability^23^. In this study, we found that female age can be used as an independent predictor of pregnancy failure after fresh cycle of Day 5 SBT. Thus it can be seen that female age alone or in combination with other clinical features (such as FSH and AFC) can be used to predict clinical pregnancy outcomes. Sperm DFI is highly sensitive and specific to detect infertility in sperm, and has higher accuracy than the conventional method of sperm evaluation^26^. It is reported that DFI have an impact on embryo quality, fertilization rates and implantation rates and^27–29^. In the present study, we found that sperm DFI can be used as an independent predictor of pregnancy failure after a fresh cycle of Day 5 SBT, which further demonstrate the importance of sperm DFI in prediction of pregnancy outcomes. Taken together, the new data presented in this study provided more evidence for us to construct a prediction model with the above two parameters.

E_2_ is an important hormone in women. Changes of serum E2 levels may affect the endometrial receptivity for embryo implantation and thus affect pregnancy outcomes. The higher the level of E_2_, the more favorable it is for embryo implantation and growth and development, and has been used to predict pregnancy outcomes^30^. Herein, we found that E_2_ was significantly increased in women with pregnancy failure compared with women with a successful pregnancy, which was consistent with previous studies. Moreover, E_2_ alone was found to be an independent predictor of pregnancy failure after fresh cycle of Day 5 SBT. Therefore, our results provide a rationalization for our use of clinical indicator E_2_ to construct predictive models. Besides, it is found that the progesterone (P)/E_2_ ratio is a better predictor than serum P alone in predicting pregnancy outcomes^31^. However, due to the limitations of various factors, we did not detect the E_2_ value of human chorionic honadotropin (hCG). Notably, serum E_2_ level on hCG day has been shown to be an independent predictor of live-birth achievement in frozen embryo transfer patients^30^. Therefore, we will continue to expand the sample size and detect hCG daily serum E2 levels to optimize our model in the future.miRNAs, a growing class of ∼ 22 nt long non-protein-coding RNAs, functioning as the universal specificity factors in post-transcriptional gene silencing, are found involved in reproductive process^32^. Hsa-miR-199a-3p is involved in inflammatory responses and embryonic gonad development^33,34^. Seminal fluidal hsa-miR-199a-5p is associated with idiopathic male infertility, endometrial receptivity and embryo implantation^35^. In IVF mouse embryos, down-regulation of hsa-miR-199a-5p lead to lower developmental potential of blastocyst^14^. Hsa-miR-99a-5p, associated with implantation, is highly expressed in germinal vesicle stage oocyte and spermatogonia of non-obstructive azoospermia patients^15,33,36^. Down-regulated hsa-miR-99a-5p is responsible for the lower developmental potential^14^. In our previous study, the above miRNAs was significantly decreased in blastocyst stages on day 5 in woman with pregnancy failure after fresh embryo transfer^16^. It is indicated that these miRNAs may be served as biomarkers for embryo quality. Therefore, these miRNAs were included as variables in the prediction model. Our results showed that these miRNAs had a potential diagnostic value for pregnancy failure after fresh cycle of Day 5 SBT. Moreover, the predictive effect of model combining 4 clinical indicators and 3 miRNAs was better than models combining single 4 clinical indicators or 3 miRNAs. It is indicated that these miRNAs can be taken into account to predict pregnancy outcome of woman after fresh cycle of Day 5 SBT.

In conclusion, the model’s prediction, using 4 clinical data and 3 miRNAs are feasible in terms of predicting pregnancy outcome of woman after fresh cycle of Day 5 SBT. This prediction model could be utilized to help the embryo transfer physician identify woman with pregnancy failure. However, there is a limitation of our study. This prediction model must be validated in a large population from geographically different areas.

## Data Availability

The datasets used and/or analysed during the current study available from the corresponding author on reasonable request.
